# Is There a Relation between Tension-Type Headache, Temporomandibular Disorders and Sleep?

**DOI:** 10.1155/2013/845684

**Published:** 2013-11-20

**Authors:** N. Caspersen, J. R. Hirsvang, L. Kroell, F. Jadidi, L. Baad-Hansen, P. Svensson, R. Jensen

**Affiliations:** ^1^Danish Headache Center, Department of Neurology, Glostrup Hospital, Nordre Ringvej 69, 2600 Glostrup, Denmark; ^2^Section of Clinical Oral Physiology, Department of Dentistry, University of Aarhus, Vennelyst Boulevard 9, 8000 Aarhus, Denmark

## Abstract

*Introduction*. Tension-Type Headache (TTH) is the most prevalent headache often associated with impaired function and quality of life. Temporomandibular Disorders (TMD) and TTH frequently coexist; characterized by pericranial tenderness and impact on daily life. We aim to apply a standardized questionnaire for TMD to characterize and analyse an eventual relation between sleep and oral health in TTH in a controlled design. *Material and Methods*. 58 consecutive TTH patients and 58 healthy controls were included. The Research Diagnostic Criteria for Temporomandibular Disorders (RDC/TMD) questionnaire, Oral Health Impact profile (OHIP) and questionnaires for sleep were applied. *Results*. TTH-patients had significantly higher pain scores (*P* < 0.001), decreased quality of life (*P* < 0.001), and higher total sleep scores (*P* < 0.001) compared to controls. *Conclusion*. For the first time we have identified a clear relation between TTH and TMD symptoms, depression, anxiety, poor sleep, and impairments of oral function in carefully classified patients. These findings indicate a close, but incomplete, overlap between TTH and TMD. Their underlying pathophysiological mechanisms need further research.

## 1. Introduction

Epidemiological studies of sleep and headache disorders point to a close connection, but the causal relations are unclear [1]. Sleep is thus reported to be the most important alleviating factor in migraine and tension-type headache (TTH) and in contrast also a trigger factor in cluster headache [1]. Likewise, disturbed sleep and lack of sleep are reported to be important trigger factors for TTH and other headaches [1]. TTH is the most prevalent headache in the adult population affecting functional and psychosocial quality of life of the patients [2]. The socioeconomic burden of TTH is thus enormous, but very little scientific attention is directed to the underlying pathophysiology and treatment strategies. 

In a large Danish longitudinal epidemiological study, we have identified disturbed sleep as a very important risk factor for poor prognosis of TTH [2], but detailed analysis of the specific relations have not yet been conducted. We haves also addressed the issue of overlaps between headaches and Temporomandibular Disorders (TMD) [2, 3] in accordance with other studies [4]. More than half of the headache patients from our tertiary headache center also had TMD diagnosis according to the Research Diagnostic Criteria for Temporomandibular Disorders (RDC/TMD) [5] and ICHD-II [6], indicating a close relationship, at least in some of the clinical manifestations of the painful conditions.

Local pain may be viewed as the most important complaint of TMD patients. In particular, pain from the masticatory or preauricular areas is frequently reported, but also headache is one of the symptoms which often occur in TMD patients [6]. In a population-based cross-sectional study of Gonçalve et al., it was noted that in patients with at least 1 TMD symptom, there was a significant overrepresentation of headache compared to TMD free individuals (56.5% versus 31.9%) [7]. Gonçalve et al. studied a group of mixed headache patients (with migraine, TTH, and/or cluster headache) and compared them to nonheadache controls with the use of the RDC/TMD. The headache group reported significantly more myofascial TMD pain and tooth clenching compared to healthy controls [7]. Overall, sleep problems, TMD and headache are also very prevalent conditions in the general population so, in theory, the relations could be spurious and coincidental rather than causal. As headache is a very broad concept covering both primary and secondary headache disorders, the precise relationship with sleep problems and with TMD is dependent on a precise headache diagnosis. 

In this study, we hypothesized that TTH patients suffer more from craniofacial pain, sleep problems, and oral health problems than healthy controls. 

Our study aim was therefore to clarify whether there was an association between TTH and self-reported TMD pain and disability and to identify potential relationships between self-reported TMD pain, sleep quality, and oral health-related quality of life in well-characterized TTH patients in comparison with healthy controls.

## 2. Material and Methods

### 2.1. Subjects

A total of 116 age- and sex-matched individuals participated in this study. We included 58 consecutive headache patients and 58 healthy controls without headache and asked them to complete 3 detailed and validated questionnaires [7–9] related to TMD, sleep, and oral function. The study participants were recruited between August 2007 and January 2011.

The headache patients were recruited from a tertiary multidisciplinary headache center in denmark (DHC) and they fulfilled the ICHD-2 criteria for frequent episodic TTH (FETTH) or chronic TTH (CTTH) [10]. They were recruited to a randomized, double-blind, placebo-controlled study of contingent electrical stimulation (CES), where the results will be published in a separate publication and the present study was part of their baseline examination.

The inclusion criteria for TTH were age between 18 and 65 years, TTH for at least 1 year before the inclusion, and completion a diagnostic headache diary for at least one month before inclusion in accordance with ICHD-II [10]. 

The exclusion criteria were more than 4 migraine days a month, medication overuse headache or head trauma, overuse of alcohol or other substance abuse, and pregnant women; cerebrovascular diseases; epilepsy or other seizure disorders. The TTH patients were not allowed to use an occlusal splint during the study and they should have kept any eventual prophylactic medication stable for at least one month before the inclusion. 

The healthy controls were recruited from the Internet or among their friends and relatives and were defined as individuals with no headache or less than 12 headache days a year; between 18 and 65 years of age; no use of medication except birth control pills; and no substance abuse. Twenty-six of our headache patients had a history of migraine and their average frequency of migraine was 2 days/month confirming that TTH is their predominant headache. The remaining 32 TTH patients reported no history of migraine. Three of the controls had also a history of migraine but no current attacks.

Both healthy controls and TTH patients gave their written informed consent and the study was approved by Local Ethical Committee as a part of the randomised controlled study (no. H-A-2007-0084).

## 3. Questionnaires

### 3.1. Research Diagnostic Criteria for Temporomandibular Disorders (RDC/TMD)

The RDC/TMD Axis II Questionnaire is a validated self-report questionnaire with 27 questions aiming to assess both functional and psychosocial aspects related to TMD pain [9].

The RDC/TMD Axis II analysis is divided into 4 parts.

Characteristic pain intensity (CPI) consists of the mean of 3 pain scores multiplied by 100 (0–100): (1) the intensity of the present pain on a visual analogue scale 0–10 (VAS) where represents 0 “no pain” and 10 represents “pain as bad as could be,” (2) the intensity of the worst pain experienced during the past six months on the same VAS, and (3) the average intensity of the pain during the past six months. The graded chronic pain scale (GCP) describes the patients' psychosocial functional level based on information from the questionnaire about, for example, the number of sick days and problems with participation of daily activities (disability points: 1–6). It rates the disabilities due to TMD pain during daily life into four grades: grade 1; low intensity of pain (CPI < 50) and low disability (less than 3 disability points): Grade 2 represents high intensity of pain (CPI > 50) and low disability (less than 3 disability points) and grades 3 (3-4 disability points) and 4 (5-6 disability points) describe increased psychosocial disability due to TMD pain regardless of the pain intensity (CPI) [9]. 

Question 15 is divided into 7 questions regarding self-reported bruxism and question 19 is a jaw disability checklist with 12 questions [11]. 

The symptom checklist (SCL) 90 is used to assess the possible involvement of depression and somatisation. On a 0–4 point scale, where 0 represents not at all, 1 represents a little bit, 2 represents moderately, 3 represents severe and 4 represents extremely severe. Furthermore all subjects indicated how much they have been bothered by pain during the past month [9]. 

### 3.2. Oral Health Impact Profile (OHIP)

OHIP is a validated questionnaire with 49 questions [8, 12] and it was developed to describe oral health and the social impact on the quality of life [8]. The questionnaire is developed from a model from Locker [13] that describes oral health [14] according to 7 dimensions: (1) experience of functional limitations; (2) pain and discomfort, (3) the impact of the orofacial problem on the mental state; and (4), (5), and (6) the perceived competence reduction in physical, psychological, and social disabilities, (7) perceived level of disability. Detailed information about OHIP has been published earlier [7].

### 3.3. Sleep/Tiredness/Snoring

The sleep quality questionnaire contains 20 questions [12]. Its purpose is to determine the quality of sleep, frequency of snoring, and tiredness during daytime [12, 15]. Detailed information about the sleep/tiredness/snoring instrument has also been published [8, 12].

## 4. Statistics

The results from the RDC/TMD Axis II (CPI, GCPS, SCL-90 depression, and somatisation scores), weighted OHIP domain scores, total weighted OHIP, score and sleep scores were analysed with unpaired *t*-tests between groups. The proportions of TTH patients and controls answering yes to RDC/TMD Axis II questions 15a-g and 19a-l were compared between groups by means of *χ* ^2^-tests. *P* values <0.05 were considered statistically significant. Data were checked for distribution of normality and parametric tests were used and the mean values are presented. Analysis for the relation between CPI, sleep, OHIP, and sleep was conducted by means of Pearson analysis of correlation. The statistical analysis was performed using the Stata 10 and Excel 2010.

## 5. Results

More than 120 questionnaires were distributed to healthy controls and 65 men and women (54%) returned the questionnaire. Seven of these were deficient and excluded from the following analysis. There were no statistically significant differences in age or gender between healthy controls and TTH patients and between responders and non-responders. The clinical baseline characteristics are described in Table 1. 

The RDC/TMD questionnaire revealed that the CPI, GCPS, and SCL-90 scores for depression and somatisation) were significantly higher in TTH patients than in control (*P* < 0.001) (Table 2).

The OHIP questionnaire revealed no significant differences between TTH patients and controls regarding psychological discomfort and physical incompetence (Table 3). TTH patients were, however, significantly more affected at the disability domain and highly physical pain, psychological incompetence, social incompetence, and handicap in the weighted total scores (*P* < 0.001). 

The total sleep score was higher in TTH patients than in healthy controls and the total numbers of hours tended also to be lower in TTH patients, although not significantly different from controls (Table 4). 

Regarding oral function, TTH patients were also significantly more affected and presented with limitations in jaw functions such as exercising, smiling/laughing and eating hard foods, and having a usual facial appearance compared to controls (Table 5).

CPI was significantly and positively correlated to frequency of headache (*r* = 0.41, *P* < 0.05) whereas no such relation between CPI and depression, anxiety, CHI, and total sleep scores could be detected. Total sleep score was significantly correlated to SCL depression (*r* = 0.52, *P* < 001) and to SCL anxiety (*r* = 0.56, *P* < 001). A less pronounced correlation was found between the total sleep score and the total weighted OHIP (*r* = 0.29, *P* < 0.05). Otherwise, no other significant correlations between sleep, CPI and OHIP were detected. 

## 6. Discussion

This study shows clearly that TTH patients are also affected by self-reported TMD pain and have impaired sleep quality and problems with oral function compared to the healthy controls. 

When we look at our analysis of the RDC/TMD evaluation in support from a priori hypothesis, it confirms the presence of a high intensity of pain in TTH patients as expected. These findings are thus in line with a prior study by Ballegaard et al. from our group [6]. It was demonstrated that TTH patients had a high prevalence of both self-reported temporomandibular pain (TMP) and TMD compared to the general population. In the paper by Glaros et al. [3], where one group with headache and one without answered the RDC/TMD questionnaire and in the headache patients, a significantly higher muscle tension in the face, the jaw, and the head was reported as well as frequent stress and more intense tooth contact. In a literature review considering TMD and headache [16], it was noted in one study that the temporal muscles contract 14 times more often during sleep in TTH patients compared to a control group without headache whereas also another well-designed study was unable to document any such variations in headache individuals [17, 18]. Our use of RDC/TMD questionnaires also confirmed the intense preponderance of self-reports of grinding, clenching, jaw-ache and discomfort that TTH patients exhibit. In daily practice, these symptoms are probably often overlooked but may be important to take into account.

Oral health impact profile shows also a very negative impact in TTH patients except from an expected impairment on physical incompetence and psychological discomfort. Likewise, the patient's oral health is significantly worse than that of the controls. It allows us to assess the relationship between physical pain and psychological burden of the individual, and both depression and anxiety were significantly correlated with the frequency of headache. This important information should thus be derived to plan the optimal, multidisciplinary treatment for the headache patients. 

This is also confirmed in a study by Moufti et al. [19] where the patients were diagnosed by RDC/TMD and the oral health impact profile questionnaire was applied by the patients and compared to a non-TMD control group. The Oral health Impact Profile and quality of life were highly impaired in TMD patients compared to non-TMD controls. This is in line with our results and underlines the importance of TMD evaluations in TTH-patients, when we investigate our patient's quality of life, their oral health and TMD issues. The study of Mitrirattanakul et al. compared patients with orofacial pain with general dental patients and also found a significant positive correlation between headache and orofacial pain [20]. Unfortunately the reported headache was not further classified in this study.

Also, sleep quality was affected for TTH patients although the normal length of sleep and hours sleep per night were not significantly different from the control group. From this, we can derive that even though the two groups slept for a similar amount of hours, the difference in quality of sleep was affecting the TTH patients in a negative way. Whether poor sleep quality leads to headache or conversely is still unclear and whether the underlying mechanisms of TTH, TMD and sleep disorders are shared by a common denominator need also clarification. Pharmacological treatment with tricyclic antidepressants (TCA) has a combined effect on sleep and headache and may thus contribute to the significant preventive effect of TCAs in TTH. Such combined mechanisms should be pursued in future treatment strategies and a detailed history of eventual sleep disorders in all TTH patients and vice versa can be recommended for providing useful information for the treatment planning and eventual prevention. Sleep quality in patients with migraine and TTH has been studied by Rains et al. [1] who concluded that multiple sleep problems or disorders have a massive negative impact on headache. Likewise another study indicated a difference between TMD patients and the so-called chronic daily headache patients in sleep quality and psychological characteristics and the authors suggested that TMD patients were more affected psychologically than chronic headache patients [21]. In line with our findings the sleep quality was also more affected in the TMD patients than in the headache patients. In this study, they used the validated sleep quality index (PSQI) a questionnaire with 17 questions developed to address posttraumatic symptoms [21]. Unfortunately a comparative study of the applied instruments is lacking and enabling a direct comparison between results. Detailed and prospective sleep analysis in properly classified TTH patients is, however, lacking.

Our study has its limitations due to the fact that the patient group was fairly severely affected and consisted mainly of CTTH patients referred to a tertiary headache center and thus not representative of TTH in the general population. Moreover, the study is cross-sectional and causal relations are thus impossible to ascertain. The advantage was the clear ICHD-diagnosis recorded prospectively in diagnostic diaries, the use of standardized instruments, and the broad baseline results. In addition, it was a clinical relevant population, a controlled study, and the patients were allowed multiple headache diagnosis as TTH as the predominant headache.

Looking at our study method with a questionnaire-based study, there may be limitations. The patient's immediate response in a questionnaire can be influenced by recall bias and the replies should be confirmed in a prospective study for both patients and controls. Such detailed recording was however out of the scope for our present study but could be of interest in future interventional studies. The advantage of a questionnaire-based study is that it probably reflects the most recent and significant problem for the patients and that the outcome can be compared with previous studies using same methodology. 

In *conclusion*, we found a clear presence of self-reported TMD symptoms, impaired sleep, and reduced oral health related quality of life in carefully classified TTH-patients compared to healthy controls whereas cause-effect relation is still uncertain. A close relation between self-reported TMD pain and headache frequency may indicate a common pain pathway whereas the sleep scores were more related to anxiety and depression. Based on these clear results, we recommend consideration of a broad treatment strategy with focus not only on the headache but also on these complicating factors. A multidimensional approach that focuses on orofacial problems, psychological reactions, and sleep quality is thus relevant and must be considered in the management of clinical TTH patients.

## Figures and Tables

**Table 1:** Demographics of patients and controls as well as headache diagnosis. Mean values and range are indicated.

	Headache patients (*N* = 58)	Controls (*N* = 58)
Age	34.5 years (18–57 years)	39.5 years (18–61 years)
Male/female	13/45	13/45
FETTH*	6	0
CTTH*	52	0
History of migraine*	26	3
Frequency (CTTH) (days/month)	28.3 (15–30 days/month)	
Frequency (f-ETTH) (days/month)	9.8 (8–12 days/month)	
Frequency total (days/month)	28.1 (8–30 days/month)	

*More than 1 headache diagnosis could be applied to the patients.

*FETTH: frequent episodic tension-type headache.

*CTTH: chronic tension type-headache.

**Table 2:** Summarized findings from RDC/TMD Questionnaire. The analysis was based on data divided into 4 groups to consider the functional and the psychosocial part of a patient's pain profile.

	Headache patients	Controls	*P* value
Characteristic pain intensity (CPI)*	55.9 ± 19.2	6.8 ± 16.3	<0.001
Graded chronic pain scale (GCP)**	2.1 ± 0.9	0.3 ± 0.5	<0.001
SCL-90 depression***	0.7 ± 0.6	0.3 ± 0.4	<0.001
SCL-90 somatisation***	0.9 ± 0.5	0.3 ± 0.4	<0.001

**Table 3:** Oral health impact profile (OHIP) in TTH patients and healthy controls. The analysis was based on data divided into the 7 dimensions and a weighted total score [13]. Weighted mean values and SDs are presented.

	Headache patients Mean/SD	Controls Mean/SD	*P* value
Disabilities	8.1 ± 6	5.9 ± 4.7	0.032
Physical pain	17.8 ± 9.9	9.1 ± 6.0	<0.001
Psychological discomfort	3.8 ± 4.6	2.97 ± 4.42	0.308
Physical incompetence	2.4 ± 4.0	1.87 ± 3.4	0.438
Psychological incompetence	6.1 ± 7.0	2.0 ± 3.8	<0.001
Social incompetence	2.6 ± 4.8	0.3 ± 1.2	<0.001
Handicap	5.6 ± 8.6	1.0 ± 2.16	<0.001
Weighted total	46.5 ± 33.3	23.2 ± 20.3	<0.001

**Table 4:** Sleep/tiredness/snoring questionnaire in TTH patients and healthy controls, describing the quality of sleep, the normal length of sleep, and hours of sleep per night. Mean values (SD) are indicated.

	Headache patients	Controls	*P* value
Total sleep score	38.4 ± 7.2	30.3 ± 4.8	<0.001
Normal length of sleep (hours)	7.2 ± 1.0	7.2 ± 0.9	0.840
Hours of sleep this night	6.8 ± 1.1	7.2 ± 1.47	0.065

**Table 5:** Self-reported oral functions from RDC in TTH patients and healthy controls. The analysis was based on data from questions 15 and 19 (abbreviated) [11].

Q. 15	Headache patients *N* (%)	Controls *N* (%)	*P* value
Clicking of the jaw, or pop	16 (28)	9 (16)	0.032
Grating or grinding noise	17 (29)	4 (7)	<0.01
Grind or clench while sleeping at night	34 (59)	8 (14)	<0.001
During the day, grindj or clench	39 (67)	8 (14)	<0.001
Morning: jaw ache or feel stiff	39 (67)	5 (9)	<0.001
Noises or ringing in ears	27 (47)	8 (14)	<0.001
Uncomfortable or unusual bite	19 (33)	4 (7)	<0.001

Q. 19, What activities does your present jaw problem prevent or limit you from doing?			

Chewing	4 (7)	3 (5)	NS
Drinking	0 (0)	2 (3)	NS
Exercising	8 (14)	1 (2)	<0.01
Eating hard foods	8 (14)	2 (3)	<0.01
Eating soft foods	0 (0)	1 (2)	NS
Smiling/laughing	7 (12)	1 (2)	<0.01
Sexual activity	1 (2)	1 (2)	NS
Cleaning teeth or face	2 (3)	2 (3)	NS
Yawning	9 (16)	4 (7)	NS
Swallowing	1 (2)	1 (2)	NS
Talking	3 (5)	1 (2)	NS
Having your usual facial appearance	7 (12)	1 (2)	<0.01

## References

[1] Rains JC, Poceta JS, Penzien DB (2008). Sleep and headaches. *Current Neurology and Neuroscience Reports*.

[2] Lyngberg AC, Rasmussen BK, Jørgensen T, Jensen R (2005). Incidence of primary headache: a Danish epidemiologic follow-up study. *American Journal of Epidemiology*.

[3] Glaros AG, Urban D, Locke J (2007). Headache and temporomandibular disorders: evidence for diagnostic and behavioural overlap. *Cephalalgia*.

[4] Anderson GC, John MT, Ohrbach R (2011). Influence of headache frequency on clinical signs and symptoms of TMD in subjects with temple headache and TMD pain. *Pain*.

[5] LeResche L, Dworkin SF, Wilson L, Ehrlich KJ (1992). Effect of temporomandibular disorder pain duration on facial expressions and verbal report of pain. *Pain*.

[6] Ballegaard V, Thede-Schmidt-Hansen P, Svensson P, Jensen R (2008). Are headache and temporomandibular disorders related? A blinded study. *Cephalalgia*.

[7] Gonçalves DAG, Bigal ME, Jales LCF, Camparis CM, Speciali JG (2010). Headache and symptoms of temporomandibular disorder: an epidemiological study: research submission. *Headache*.

[8] Allen PF, McMillan AS, Walshaw D, Locker D (1999). A comparison of the validity of generic- and disease-specific measures in the assessment of oral health-related quality of life. *Community Dentistry and Oral Epidemiology*.

[9] Dworkin SF, Sherman J, Mancl L, Ohrbach R, LeResche L, Truelove E (2002). Reliability, validity, and clinical utility of the research diagnostic criteria for temporomandibular disorders axis II scales: depression, non-specific physical symptoms, and graded chronic pain. *Journal of Orofacial Pain*.

[10] (2004). * The International Classification of Headache Disorders*.

[11] Dworkin SF, LeResche L (1992). Research diagnostic criteria for temporomandibular disorders: review, criteria, examinations and specifications, critique. *Journal of Craniomandibular Disorders*.

[12] Fransson AMC, Tegelberg A, Leissner L, Wenneberg B, Isacsson G (2003). Effects of a mandibular protruding device on the sleep of patients with obstructive sleep apnea and snoring problems: a 2-year follow-up. *Sleep & Breathing*.

[13] Locker D (1988). Measuring oral health: a conceptual framework. *Community Dental Health*.

[14] Hans Gjørup, Svensson P OHIP-(D), en danskversion af Oral Health Impact Profile.

[15] Jadidi F, Nørregaard O, Baad-Hansen L, Arendt-Nielsen L, Svensson P (2011). Assessment of sleep parameters during contingent electrical stimulation in subjects with jaw muscle activity during sleep: a polysomnographic study. *European Journal of Oral Sciences*.

[16] Laibovitz BM (2006). *Temporomandibular Disorders and Headache: A Review of the Literature*.

[17] Jensen R, Olesen J (1996). Initiating mechanisms of experimentally induced tension-type headache. *Cephalalgia*.

[18] Steele JG, Lamey PJ, Sharkey SW, Smith GM (1991). Occlusal abnormalities, pericranial muscle and joint tenderness and tooth wear in a group of migraine patients. *Journal of Oral Rehabilitation*.

[19] Moufti MA, Wassell RW, Meechan JG, Allen PF, John MT, Steele JG (2011). The Oral Health Impact Profile: ranking of items for temporomandibular disorders. *European Journal of Oral Sciences*.

[20] Mitrirattanakul S, Merrill RL (2006). Headache impact in patients with orofacial pain. *Journal of the American Dental Association*.

[21] Vazquez-Delgado E, Schmidt JE, Carlson CR, DeLeeuw R, Okeson JP (2004). Psychological and sleep quality differences between chronic daily headache and temporomandibular disorders patients. *Cephalalgia*.

